# Gene expression profiling via LongSAGE in a non-model plant species: a case study in seeds of *Brassica napus*

**DOI:** 10.1186/1471-2164-10-295

**Published:** 2009-07-03

**Authors:** Christian Obermeier, Bashir Hosseini, Wolfgang Friedt, Rod Snowdon

**Affiliations:** 1 Justus Liebig University Giessen, Department of Plant Breeding, Heinrich-Buff-Ring 26-32, 35392 Giessen, Germany

## Abstract

**Background:**

Serial analysis of gene expression (LongSAGE) was applied for gene expression profiling in seeds of oilseed rape (*Brassica napus *ssp. *napus)*. The usefulness of this technique for detailed expression profiling in a non-model organism was demonstrated for the highly complex, neither fully sequenced nor annotated genome of *B. napus *by applying a tag-to-gene matching strategy based on *Brassica *ESTs and the annotated proteome of the closely related model crucifer *A. thaliana*.

**Results:**

Transcripts from 3,094 genes were detected at two time-points of seed development, 23 days and 35 days after pollination (DAP). Differential expression showed a shift from gene expression involved in diverse developmental processes including cell proliferation and seed coat formation at 23 DAP to more focussed metabolic processes including storage protein accumulation and lipid deposition at 35 DAP. The most abundant transcripts at 23 DAP were coding for diverse protease inhibitor proteins and proteases, including cysteine proteases involved in seed coat formation and a number of lipid transfer proteins involved in embryo pattern formation. At 35 DAP, transcripts encoding napin, cruciferin and oleosin storage proteins were most abundant. Over both time-points, 18.6% of the detected genes were matched by *Brassica *ESTs identified by LongSAGE tags in antisense orientation. This suggests a strong involvement of antisense transcript expression in regulatory processes during *B. napu*s seed development.

**Conclusion:**

This study underlines the potential of transcript tagging approaches for gene expression profiling in *Brassica *crop species via EST matching to annotated *A. thaliana *genes. Limits of tag detection for low-abundance transcripts can today be overcome by ultra-high throughput sequencing approaches, so that tag-based gene expression profiling may soon become the method of choice for global expression profiling in non-model species.

## Background

Seed developmental processes of plants are complex and follow coordinated gene expression programs. Some genes are exclusively expressed during seed development [1]. Understanding the genetics of developmental processes and regulatory networks involved in embryogenesis and seed development is crucial for improvement of seed quality in crop plants. Gene expression during seed development has been studied intensively in the model plant *Arabidopsis thaliana *using mutagenesis [2,3] and microarray analyses (e.g., [3,4]). These studies revealed major gene expression changes during seed filling and desiccation, along with distinct expression patterns related to carbohydrate metabolism, lipid biosynthesis and storage protein accumulation. However, the regulatory mechanisms that ensure the proper execution of seed development in *A. thaliana *and other plants remain largely unknown. Furthermore, generalisation of findings regarding gene regulation from model to crop plants is difficult because most major crops have considerably more complex genomes.

Oilseed rape (*Brassica napus *ssp. *napus*), the closest major crop relative of *A. thaliana*, is the world's second most important oilseed crop. The high-value nutritional oil is also an excellent substrate for biodiesel production, whereas the protein-rich seed meal remaining after oil extraction is a valuable livestock feed. Oligonucleotide microarrays constructed for *A. thaliana *have been used in the past for expression profiling in *B. napus*, but have not provided optimal signal intensity and reproducibility [4-7]. Recently the total number of ESTs from *Brassica *species deposited in public databases has risen dramatically to more than 800,000 entries with about 280,000 from seed developmental stages. 67,000 ESTs from seed developmental stages have been used to develop a *B. napus *cDNA microarray for analysis of seed gene expression patterns [7].

An alternative for accurate, quantitative global expression profiling is serial analysis of gene expression (SAGE; [8]), which in contrast to microarray hybridization allows the detection of new transcripts. SAGE is an expression profiling technique that simultaneously measures the levels of thousands of genes expressed in a given tissue. The method is based on the excision of short tags from poly A+ RNAs and end-to-end ligation of ditags to form high molecular weight concatemers. This allows cost-effective high-throughput cloning and sequencing of concatemers. Matching of tags to genomic sequences is a critical step in SAGE data analysis, and normally this requires the availability of high quality genome annotation data [9]. SAGE was first used for quantification of gene expression in human using 13–15 bp tags [8]. Modifications of the original SAGE protocols producing 21 bp tags (LongSAGE; [9]) and 26 bp tags (SuperSAGE; [10]) have been developed to enable more efficient and unambiguous tag-to-gene assignment in higher organisms with more complex transcriptomes. SAGE is commonly used in animal genomics, but has been increasingly used in plant species and tissues [11]. The intention of the present study was to adapt the LongSAGE technique for analysis of global gene expression in *B. napus *and other similarly complex polyploid plant genomes where the complete genome sequence and annotation are not yet available. A data processing pipeline was adapted by matching *B. napus *tags via *Brassica *ESTs to annotated *A. thaliana *gene loci, including detection of tags matching in sense and antisense orientation.

## Results

### *B. napus *seed LongSAGE libraries

Two LongSAGE libraries were produced from *B. napus *seeds harvested at 23 and 35 DAP, respectively. A total of 3,136 clones from the 23 DAP library yielded 34,984 ditags (22 tags/clone), while 3,168 sequence traces from the 35 DAP library yielded 15,919 ditags (10 tags/clone) (Table 1). The relatively low average number of tags per clone resulted from a high number of cloned empty vector plasmids (28.2% and 60.3% of all high-quality reads for the 23 DAP and 35 DAP libraries, respectively). The vector plasmid pZErO (Invitrogen) was designed to prevent cloning of empty vector by using a LacZa-ccdB fusion cDNA insert that should destroy bacteria containing only the vector. However, as described by other authors (e.g. [12]) we found that pZErO could efficiently re-ligate without containing inserts. Thus, PCR screening of plasmid libraries for clones containing an empty vector might considerably reduce total Sanger sequencing costs and increase total tag sample sizes in LongSAGE transcription profiling experiments.

**Table 1:** Summary of LongSAGE tags sequenced from *B. napus *seeds at 23 and 35 days after pollination (DAP).

	**23 DAP library**		**35 DAP library**		**Combined data**	
	
**Category**	**Unique sequences**	**All sequences**	**Unique sequences**	**All sequences**	**Unique sequences**	**All sequences**
Total extracted ditags	-	34,984	-	15,919	-	50,903
Rejected ditags (Phred score ≤ 20^a^)	-	10,685	-	3,306	-	13,991
Accepted ditags (Phred score > 20^a^)	23,996	24,299	11,846	12,613	35,842	36,912
Total extracted tags	24,655	48,534	12,714	25,198	37,369	73,732

Accepted tags (excluding polyA tags)	24,654	48,514	12,713	25,183	37,367	73,697

**Copy number of accepted tags**						

1	18,183 (73.8.%)	18,183 (37.5%)	9,741 (76.6%)	9,741 (38.7%)		
2–5	5,329 (21.6%)	14,320 (29.5%)	2,510 (19.7%)	6,596 (26.2%)		
6–20	1,016 (4.1%)	9,450 (19.5%)	406 (3.2%)	3,668 (14.6%)		
21–99	118 (0.5%)	4,437 (9.1%)	46 (0.4%)	1,997 (7.9%)		
>100	8 (0.03%)	2,124 (4.4%)	9 (0.07%)	3,181 (12.6%)		

^a ^Using Sage-phred.pl, including duplicated ditags

Table 1 summarises the numbers of accepted and rejected tags in the two libraries. In total, 73,697 tags comprising 37,367 unique tags were extracted from both libraries. Valid tags from 23 DAP and 35 DAP were deposited in the Gene Expression Omnibus (GEO) public domain under accession number GSE14313. The total number of accepted tags from the 23 DAP library was 48,514 comprising 24,654 unique tags. The total number of accepted tags from the 35 DAP library was 25,183 tags comprising 12,713 unique tags. 67.0% and 64.9% of the accepted total tags were represented by five or less copies in the 23 DAP and 35 DAP libraries, respectively.

### Matching *B. napus *tags to the *A. thaliana *proteome via *Brassica *ESTs

Table 2 summarises the results from the tag-to-gene matching analysis of the two libraries. Initially, all ESTs from a redundant database consisting of 834,732 *Brassica *EST entries (from the A and C genome brassicas *B. napus*, *B. oleracea*, *B. rapa*) were oriented by blastx alignment against the *A. thaliana *TAIR7 proteome (cut-off value 1e-6). Virtual tags were then extracted from all successfully oriented ESTs and assigned via these ESTs to the annotated *A. thaliana *proteins. A total of 3,563,603 virtual 21 bp tags were extracted from all positions of the ESTs. From the 32,395 different observed tags, 15,955 (49.3%) were matched under the applied conditions to *Brassica *A or C genome ESTs (Table 2). Matching success was reduced about 30% for singleton tags compared to tags with counts ≥ 2. For tags with counts ≥ 2 the matching success amounted to about 80% (Table 2). This discrepancy might be due to the reduced numbers of ESTs in the database derived from transcripts expressed at low levels, compared to transcripts expressed at high levels within *B. napus *seeds. Broadly different estimates exist on the proportion of errors within tag sequences caused by experimental artefacts, ranging from 1.67% to 15.6% of LongSAGE tags [13,14] with at least one erroneous base-pair introduced by sequencing. However, the singleton tag category might contain a higher proportion of tags with sequencing artefacts, also contributing to the different ratio of successfully matched tags in the singleton and ≥ 2 count categories. 12,728 tags from library 23 DAP were matched to 7,208 of 20,462 *Brassica *EST-matched *A. thaliana *proteins. 7,232 tags from library 35 DAP were matched to 4,875 *A. thaliana *proteins (Table 2).

**Table 2:** Summary of LongSAGE tag-to-gene mapping data for two libraries from *B. napus *seeds at 23 and 35 days after pollination (DAP).

Analysis including all singleton tags	23 DAP library	35 DAP library	combined data
Number of Brassica ESTs matched to the *A. thaliana *proteome	834,732		
Number of *A. thaliana *genes matched by Brassica ESTs	20,462		

Number of tags accepted for processing	24,654	12,713	32,395
Number of Brassica ESTs matched by tags	256,420	214,981	301,180
Total number of accepted tags matched to Brassica ESTs	12,728 of 24,654 (51.6%)	7,232 of 12,713 (56.9%)	15,955 of 32,395 (49.3%)
Number of accepted tags not matched to any Brassica EST	11,926 of 24,654 (48.4%)	5,481 of 12,713 (43.1%)	16,400 of 32,395 (50.6%)

Number of accepted singleton tags matched to Brassica ESTs	7,615 of 18,183 (41.9%)	4,819 of 9,741 (49.5%)	11,421 of 26,498 (43.1%)
Number of accepted tags with ≥2 counts matched to Brassica ESTs	5,513 of 6,471 (85.2%)	2,413 of 2,972 (81.2%)	6,010 of 7,727 (77.8%)

Number of *A. thaliana *genes matched via Brassica ESTs and tags	7,208	4,875	8,255

Number of *A. thaliana *genes matched and oriented via Brassica ESTs and tags (putative chimeric ESTs removed)	4,478	3,005	5,120

Number of genes matched in both directions	1,125 (25.1%)	560 (18.6%)	1,453 (28.4%)
Number of genes matched in sense orientation exclusively	3,057 (68.3%)	2,242 (74.6%)	3,358 (69.5%)
Number of genes matched in antisense orientation exclusively	296 (6.6%)	203 (6.8%)	309 (6.1%)

### Comparison of LongSAGE and Real-time RT-PCR analysis

A good correlation was observed between expression levels estimated by LongSAGE and Real-time RT-PCR for most of the selected genes (Table 3), although some minor differences in estimated fold-change were observed (e.g. for CCR1). This could arise from the more selective specificity of Real-time RT-PCR for transcripts derived from closely-related gene loci, since specific RT-PCR primers were designed from contigs of *B. napus *ESTs putatively derived from different *B. napus *gene loci. Furthermore, Real-time RT-PCR may be more sensitive in detecting low abundant transcripts than LongSAGE under the experimental conditions and sequencing depth applied in the present study.

**Table 3:** Comparison of fold-changes for selected genes between 23 and 35 DAP in *B. napus *seeds measured by LongSAGE and Real-time RT-PCR.

***A. thaliana *gene locus**	**Gene**	**Cumulated counts,35:23 DAP LongSAGE (non-normalized)**	**Fold-change 35:23 DAP LongSAGE (normalized)**	**Fold-change 35:23 DAP Real-time RT-PCR**	***B. napus *EST contigs assembled by CAP3**
AT2G40890	Coumarate 3-hydroxylase (C3H)	2:2	1.9	2.0 4.1	Contig 1 from 5 ESTs Contig 2 from 2 ESTs

AT5G48930	Hydroxycinnamoyl-Coenzyme A shikimate/quinate hydroxycinnamoyl-transferase (HCT)	5:3	3.2	2.3	1 EST

AT1G15950	Cinnamoyl-CoA reductase 1 (CCR1)	2.2:1	4.3	0.9	Contig from 4 ESTs

AT2G23910	Cinnamoyl-CoA reductase-related (CCR1-related)	0:0	-	1.2 1.7	Contig 1 from 22 ESTs Contig 2 from 13 ESTs

AT3G19450	Cinnamoyl alcohol dehydrogenase (CAD1)	8:9.5	1.6	2.1	Contig 3 from 4 ESTs

AT4G27140 AT4G27150 AT4G27160 AT4G27170	2S seed storage proteins (napin)	669.1:3.4 560.1:1.9 575.3:1.9 636.5:7.9	374.1 564.4 579.6 155.0	1224	Contig from SAGE primer-amplified cloned 3'-terminal napin gene fragments

Primers for Real-time RT-PCR were designed using assembled *B. napus *ESTs matched to *A. thaliana *proteins using blastx with *e*-values greater than 1e-6.

### Transcript diversity

Average tag frequencies for the two analysed seed libraries differed significantly (Table 1). Whereas tags with medium frequency (2–20) were more abundant in the 23 DAP library, the 35 DAP library was considerably more enriched for high copy-number tags with a frequency ≥100. Nine unique tags with ≥100 counts each represented 3.2% of the total tag number at 35 DAP, whereas the 8 tags with ≥100 counts each at 23 DAP comprised only 2.1% of the total tags. Accordingly, the relative diversity of tags was greater at 23 DAP than at 35 DAP. We detected 48% (7,208 compared to 4,875) more expressed genes at 23 DAP than at 35 DAP (Table 2), which is consistent with the number of different tags observed in each of the two libraries.

To validate the LongSAGE method and estimate the diversity of transcripts, LongSAGE tags derived from selected tags with 2–32 counts in the 35 DAP library were used as primers for RT-PCR in combination with anchored oligo-dT primers. In 17 out of 17 cases a PCR product was amplified and sequenced that aligned in blastn analysis with *Brassica *or *A. thaliana *ESTs or cDNAs. A further validation was performed with 9 different LongSAGE tags that had 3 to 325 counts in the 35 DAP library and matched members of the *A. thaliana *2S storage gene family or ESTs/cDNAs from the *Brassica *napin gene family. In all cases the PCR amplification products, ranging from 75 to 457 bp in length, were related to *Brassica *napin genes or other protease inhibitor/seed storage/lipid transfer protein (LTP) family protein genes (56 to 100% identity). A high diversity of 3' termini of clones were obtained using the same napin tag primer, with up to 14% divergence, within 200–400 bp of the 3' untranslated region (UTR), from up to 8 clones per amplicon. Interestingly, diverse 3'-UTR sequences were also obtained using different napin tag primers, with up to 30% divergence, within 200 bp of the 3'-UTR, from 16 clones. These observations suggest that a high number of distinct protease inhibitor/seed storage/lipid transfer protein (LTP) family protein genes or transcript variants are expressed in *B. napus *seeds. Also these data support the increasing recognition of the high complexity and variability of 3' UTR regions in genes from higher organisms [13,15,16].

Based on the cDNA library production using polyA+ trapped mRNA, LongSAGE tags are expected to match at the anchoring enzyme site closest to the 3' end of full-length ESTs expressed in sense orientation (canonical position). However, only 95.3% of the matched tags in the sense EST dataset were matched in the last canonical position. There are numerous possible explanations for the high frequency of tags matching a non-canonical position. In particular, they could arise from (a) cloning artefacts caused by partial digestion with the anchoring enzyme *Nla*III during SAGE library preparation, (b) alternative transcript variants with varying length derived from one gene locus, or (c) EST cloning artefacts creating chimeric EST molecules derived from two or more different genes. Also, due to partial EST sequences present in the dataset, a non-canonical position might be falsely annotated to the canonical position. Except in the third case, non-canonical tags are still valid tags for accurate annotation. We attempted to reduce annotation artefacts (c) by removing putative chimeric ESTs from analysis (see section 'Genes matched in sense and antisense orientation' for details). This increased the ratio of tags matched to the canonical position of oriented *Brassica *ESTs from 95.3% to 98.3%. However, even after filtering out putative experimental artefacts using this approach, only 77% of all 243,528 ESTs matched by tags in the canonical position are matched exclusively at this position. Instead, 23% of these individual ESTs are additionally matched by tags at other non-canonical positions (up to the 9^th ^before last position), suggesting that the usage of alternative transcripts (e.g. by alternative polyadenylation) is a common mechanism. In other words, a high diversity exits in the 3' UTR of many expressed transcripts, as has been described before in humans [13]. No information exists about transcription start points for polyA+ containing transcripts expressed in antisense orientation. Therefore different proportions of matched antisense tags might be expected at canonical and non-canonical positions. In contrast to the tags matched in sense orientation, only 65.5% of tags were matched to canonical positions for the tags matched in antisense orientation.

### Comparison of the most abundantly expressed tags and assigned genes

Tag-to-EST matches based on 15,955 tags were assigned to a total of 8,255 *A. thaliana *gene loci at both time-points (Table 2). About 85% of the tags (13,641) were assigned exclusively to a single *A. thaliana *gene locus. The other 15% of the tags matched 2 to 45 loci, with some tags matching more than 10 genes exhibiting a polyA-like homopolymer composition. Due to the positioning of the anchoring enzyme restriction site close to the polyA tail in some transcripts, these contained stretches with up to 16 adenosine bases derived from phylogenetically unrelated genes. Other tags matching more than 2 genes were clearly derived from groups of phylogenetically related genes. For example, the tag CATGAACAGTTTCATCAACGA matched to six different members of the histone gene family (AT2G37470, AT3G53650, AT5G22880, AT5G59910, AT1G07790 and AT5G02570).

On the other hand, some *A. thaliana *loci were matched by up to 96 different *B. napus *tags via the *Brassica *ESTs. Moreover, some loci were not only matched by different tags, but these tags also matched at different positions within one particular matched EST molecule. These multiple matches underline the complex paleopolyploid structure of the *B. napus *genome in comparison to *A. thaliana *[17]. For example, the 4 *A. thaliana *gene loci of the 2S seed storage protein family were matched by 47 different tags, including 9 of the 30 most abundant tags, at 35 DAP. Due to this complexity, a three-step procedure was applied for calculating average values of the relative abundance of gene expression, based on the tag-to-gene matching results for each single *A. thaliana *gene locus. In a first step, the measured counts were evenly distributed to the matched gene loci if a tag matched more than one gene locus. In a second step, tag counts were added together if different tags matched the same gene locus. In a final step the summed tag counts for each gene locus were normalized to a total tag count of 1,000,000 for both libraries and the relative abundances were calculated.

Average cumulative counts and frequencies for the 20 most abundantly expressed genes at 23 and 35 DAP are shown in Table 4 and 5, respectively. The most abundant transcripts at 23 DAP, with 1.59% of all matched tag counts, correspond to the *A. thaliana *senescence-associated cysteine-type protease SAG12 (AT5G45890), which was also among the 20 most highly-expressed genes at 35 DAP. Two protease inhibitor/seed storage/lipid transfer family proteins (AT5G38195, AT1G48750) were also among the 20 most highly expressed genes at both time-points. Other highly expressed transcripts at 23 DAP are a number of catalytic enzymes and other genes that are involved in diverse biological processes. At 35 DAP, transcripts related to the four closely-related *A. thaliana *2S seed storage protein genes showed the highest counts, with a cumulative abundance of about 10%. Other highly expressed transcripts at 35 DAP are related to other storage proteins (cruciferin, oleosin), protease inhibitor or lipid transfer proteins and genes involved in fatty acid biosynthesis. For many of the highly expressed genes several different tags were found (e.g. 44 for AT5G45890 at 23 DAP) matching to a large number of different ESTs (e.g. 5,452 for AT5G45890 at 23 DAP) that were aligned to a particular *A. thaliana *gene at <1e-6.

**Table 4:** Summary of the 20 most abundant genes expressed in *B. napus *seeds at 23 days after pollination (DAP) based on LongSAGE data analysis.

Rank	*A. thaliana *locus	No. of matched *Brassica *ESTs at 1e-6	No. of different tags matched to *Brassica *ESTs	Mean tag count per locus	Frequency of tag counts (%, normalized)	Description of matched *A. thaliana *gene locus
1	AT5G45890	4,452	44	772	1.590	SAG12 (SENESCENCE-ASSOCIATED GENE 12); cysteine-type peptidase
2	AT5G50260	109	15	320	0.660	cysteine proteinase, putative
3	AT3G48350	14	7	299	0.615	cysteine proteinase, putative
4	AT5G38195	1,640	36	263	0.541	protease inhibitor/seed storage/lipid transfer protein (LTP) family protein
5	AT1G48750	8	4	209	0.430	protease inhibitor/seed storage/lipid transfer protein (LTP) family protein
6	AT3G20210	630	13	156	0.323	DELTA-VPE (delta vacuolar processing enzyme); cysteine-type endopeptidasee
7	AT2G12465	160	11	134	0.275	LCR50 (Low-molecular-weight cysteine-rich 50)
8	AT4G32110	852	20	127	0.263	transferase, transferring glycosyl groups
9	AT3G04120	814	30	124	0.256	GAPC (GLYCERALDEHYDE-3-PHOSPHATE DEHYDROGENASE C SUBUNIT)
10	AT5G37474	35	10	120	0.247	Encodes a defensin-like (DEFL) family protein
11	AT2G14846	191	13	118	0.244	protease inhibitor/seed storage/lipid transfer protein (LTP) family proteinperoxidise
12	AT4G21960	1,312	16	118	0.243	PRXR1 (peroxidase 42); peroxidase
13	AT5G60390	2,771	30	113	0.233	elongation factor 1-alpha/EF-1-alpha
14	AT4G32105	79	13	109	0.225	Galactosyltransferase
15	AT1G58055	23	5	100	0.206	Encodes a defensin-like (DEFL) family protein
16	AT1G04645	502	11	97	0.200	self-incompatibility protein-related
17	AT1G05850	426	11	96	0.198	POM1; chitinase
18	AT1G08830	410	14	95	0.196	CSD1 (copper/zinc superoxide dismutase 1)
19	AT4G12960	351	6	93	0.191	gamma interferon responsive lysosomal thiol reductase family protein/GILT
20	AT4G21650	53	8	92	0.189	subtilase family protein

**Table 5:** Summary of the 20 most abundant genes expressed in *B. napus *seeds at 35 days after pollination (DAP) based on LongSAGE data analysis.

Rank	*A. thaliana *locus	No. of matched *Brassica *ESTs at 1e-6	No. of different tags matched to *Brassica *ESTs	Mean tag count per locus	Frequency of tag counts (%, normalized)	Description of matched *A. thaliana *gene locus
1	AT4G27140	16,413	96	669	2.657	2S seed storage protein 1/2S albumin storage protein/NWMU1-2S albumin 1
2	AT4G27170	6,691	67	637	2.528	2S seed storage protein 4/2S albumin storage protein/NWMU2-2S albumin 4
3	AT4G27160	2,402	34	575	2.284	2S seed storage protein 3/2S albumin storage protein/NWMU2-2S albumin 3
4	AT4G27150	249	24	560	2.224	2S seed storage protein 2/2S albumin storage protein/NWMU2-2S albumin 2
5	AT4G28520	126	13	195	0.776	CRU3 (CRUCIFERIN 3)
6	AT3G27660	1,226	15	183	0.728	OLEO4 (OLEOSIN4)
7	AT5G38195	1,640	36	161	0.640	protease inhibitor/seed storage/lipid transfer protein (LTP) family protein
8	AT4G25140	1,119	15	146	0.580	OLEO1 (OLEOSIN1)
9	AT5G44120	1,112	17	143	0.567	CRA1 (CRUCIFERINA); nutrient reservoir
10	AT5G45890	4,452	44	143	0.566	SAG12 (SENESCENCE-ASSOCIATED GENE 12); cysteine-type peptidase
11	AT1G03880	1,791	12	138	0.549	CRU2 (CRUCIFERIN 2); nutrient reservoir
12	AT1G48750	8	4	136	0.538	protease inhibitor/seed storage/lipid transfer protein (LTP) family protein
13	AT1G79870	5	3	123	0.487	oxidoreductase family protein
14	AT1G12920	1	2	113	0.447	ERF1-2 (EUKARYOTIC RELEASE FACTOR 1-2); translation release factor
15	AT5G35530	401	6	102	0.406	40S ribosomal protein S3 (RPS3C)
16	AT3G12580	31	8	102	0.405	HSP70 (heat shock protein 70); ATP binding
17	AT3G01570	170	6	99	0.393	glycine-rich protein/oleosin
18	AT1G69830	1	2	97	0.385	AMY3/ATAMY3 (ALPHA-AMYLASE-LIKE 3); alpha-amylase
19	AT3G05020	401	14	86	0.340	ACP1 (ACYL CARRIER PROTEIN 1)
20	AT5G09440	31	3	82	0.326	phosphate-responsive protein, putative

### Differentially expressed genes

620 tags showed differential expression at *p *≤ 0.05 between 23 and 35 DAP. These represent only 1.7% of the accepted unique tags, but 21.7% of all normalized tag counts. 498 of 620 (80.3%) differentially expressed unique tags were successfully matched to 490 *A. thaliana *genes (5.9% of all 8,255 matched genes, Table 2), and these differentially expressed genes were matched by 18.2% and 38.1% of all matched tags in 23 DAP and 35 DAP libraries, respectively (see additional file 1: 490_differentially_expressed_genes.xls). This indicates that, in the 35 DAP library but not in the 23 DAP library, many of the differentially expressed transcripts are highly abundant. Of these 490 genes, 194 genes were upregulated at 23 DAP and 296 genes were upregulated at 35 DAP. The 20 most abundant differentially expressed genes at 23 DAP include 6 genes encoding proteases, 5 genes coding for protease inhibitor/seed storage/lipid transfer protein (LTP) family proteins and 2 genes coding for defensin-like family proteins. SAG12 is the most abundant differentially expressed gene at 23 DAP with 1.591% and is down-regulated about 3-fold at 35 DAP with 0.566% total abundance. The 20 most abundant differentially expressed genes at 35 DAP cover a narrower range of genes coding for 2S seed storage proteins, oleosins, cruciferins and protease inhibitor proteins. The 5 most abundant differentially expressed genes are the 2S seed storage proteins 1 to 4 and cruciferin 3. The 2S seed storage protein 1 is the most abundant differentially expressed gene with 2.657% abundance and is up-regulated 374-fold compared to time-point 23 DAP. From all 490 genes showing a significantly different expression level at the two time-points, 23 were exclusively expressed at 23 DAP and 54 at 35 DAP (see additional file 1: 490_differentially_expressed_genes.xls). A number of cases were observed where different matched *A. thaliana *genes with similar functional annotations were expressed at the two different time-points, e.g. different *A. thaliana *genes belonging to the large cysteine protease and lipid transfer protein families were exclusively expressed at 23 DAP and 35 DAP, respectively. In contrast to cruciferin A (CRA1) and cruciferin 3 (CRU3), cruciferin 2 (CRU2) was exclusively expressed at 35 DAP.

Expression data produced by microarray hybridization for 79 developmental stages of *A. thaliana *were downloaded from EBI ArrayExpress (accession number E-AFMX-9, [18]) and expression profiles were compared with expression profiles obtained by LongSAGE for *B. napus *seeds using hierarchical cluster analysis and Spearman's rank correlations according to Lu et al. [19]. Both *B. napus *SAGE data sets showed weak correlations with expression value estimates from *A. thaliana *microarray data sets with the highest correlation for the seed developmental stages ATGE_77 (0.522 for 23 DAP) and ATGE_78 (0.440 for 35 DAP). Sample ATGE_77 was derived from *A. thaliana *seed stage 4, early to late heart stage embryos and sample ATGE_78 from seed stage 5, late heart to mid torpedo embryos. Embryos dissected from sample 23 DAP were classified to be at the mid torpedo stage and from sample 35 DAP to be at the early curled cotyledon stage. Genes that were differentially expressed between two different *A. thaliana *seed stages (ATGE_77 and ATGE_78) were identified at different stringencies and compared with the 490 genes identified to be differentially expressed between 23 DAP and 35 DAP in *B. napus *as described above. From the 490 genes identified by LongSAGE 76 (15%) were not represented on the microarray chip. From the remaining 414 genes a maximum of 29% (122) overlapping genes were detected that were mainly highly abundant genes (e.g. 2S seed storage proteins). The percentage of overlapping genes identified here between the two different crucifer species during morphological similar seed developmental stages appears to be high when taking into account that the correlation of microarray hybridization and LongSAGE data analysis has been found to be low (below 0.5) even when using the same RNA preparations and LongSAGE has been characterized to be more efficient at identifying differentially expressed tags than microarray technology [19].

### Gene ontology enrichment analysis

**Figure 1:**
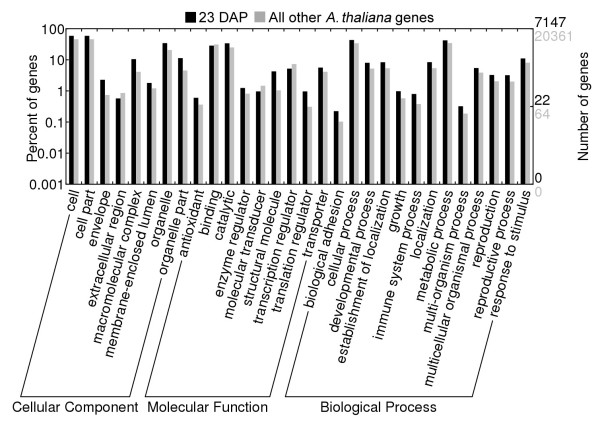
**Statistically enriched Gene Ontology terms level 2 at *p *≤ 0.05 in *B. napus *seeds for LongSAGE tag-matched *A. thaliana *gene loci at 23 DAP, compared with level 2 GO terms for all other *A. thaliana *gene loci**. The plot was produced using the online tool WEGO (Ye *et al*., 2006).

A total of 7,208 of the 8,255 matched genes were expressed at 23 DAP, while 4,875 genes were expressed at 35 DAP (Table 2). 3,828 of 8,255 genes (46.4%) were expressed at both time-points. Figure 1 gives a comparison of GO categories that are statistically over-represented for the genes expressed at 23 DAP compared to all other *A. thaliana *genes. From the 490 genes matched by statistically significant differentially expressed tags between 23 and 35 DAP, 194 were up-regulated at 23 DAP and 296 were up-regulated at 35 DAP. Statistically enriched GO terms found for the differentially expressed genes either up-regulated at 23 or up-regulated at 35 DAP were calculated using GOEAST [20] and details of the results are provided in additional file 2: Enriched_GO_terms_490_differentially_expressed_genes.xls. Within the category 'Biological Process', the GO terms 'developmental process', 'localization' and 'metabolic process' are enriched at both time-points. Generally, GO terms that are statistically enriched at the highly general information level 2 are similar for the two time-points. Comparison of statistically enriched GO terms for 490 differentially expressed genes at 23 and 35 DAP reveals their involvement in seed maturation, regulation of meristem organization and photomorphogenesis, seed coat development, water and fluid transport, cell-cell signalling, cell wall modification and glycerolipid, neutral lipid, acylglycerol, triglyceride, and carbohydrate metabolic processes at 23 DAP, and in protein processing, protein targeting, photosynthesis, fatty acid biosynthesis, lipid localization, storage and lipid metabolic processes at 35 DAP.

### Genes matched in sense and antisense orientation

In the 23 DAP library, 6 of the 30 most abundant tags matched in sense as well as in antisense orientation to the *Brassica *EST dataset, oriented based on the *A. thaliana *proteome. The same was true for 10 of the 30 mosXt abundant tags at 35 DAP. In most of these cases 500 to 1,500 ESTs were matched by a single tag in one direction, whereas only 1 to 13 ESTs were matched by the same tag in the opposite direction. A detailed analysis of these matches revealed that in all cases the smaller fraction of matched ESTs were chimeric sequences composed of two regions, derived from different gene loci, that aligned to the *A. thaliana *proteome or to *B. napus *full-length cDNAs in different orientations. These apparently chimeric ESTs may represent cloning artefacts derived from tail-to-tail or head-to-head ligations during cDNA preparation for EST library production. In some cases a putative chimeric composition was also found for individual ESTs, with two different regions derived from different genes being aligned in sense and antisense orientation to the *A. thaliana *proteome or to oriented *B. napus *full-length cDNAs. To remove these putative chimeric ESTs from our analysis, tag-to-EST-to-*A. thaliana *gene locus matches were only accepted if at least 4 tag-matched *Brassica *ESTs matching a particular *A. thaliana *locus were found. In addition, tag-to-EST-to-gene matches of particular tags matching in sense as well as in antisense orientation were only included in the analysis if the frequency of ESTs matched in one direction was at least 1% of the frequency of ESTs matched in the other direction. After removal of putative chimeric ESTs, the number of accepted tags that were successfully matched via *Brassica *ESTs to *A. thaliana *genes was reduced strongly from 8,255 to 5,120 (Table 2), due to the exclusion of many low-copy ESTs from the analysis. The occurrence of chimeric ESTs in databases has been documented previously. For example, Hillier *et al*. [21] found chimeric ESTs in a dataset of 280,000 human ESTs at a frequency of up to 1.04%. The strategy we applied resulted in removal of all 72 chimeric ESTs that were identified by manual analysis of the 47,446 ESTs matched by the 30 most abundant tags in the two LongSAGE libraries.

**Figure 2:**
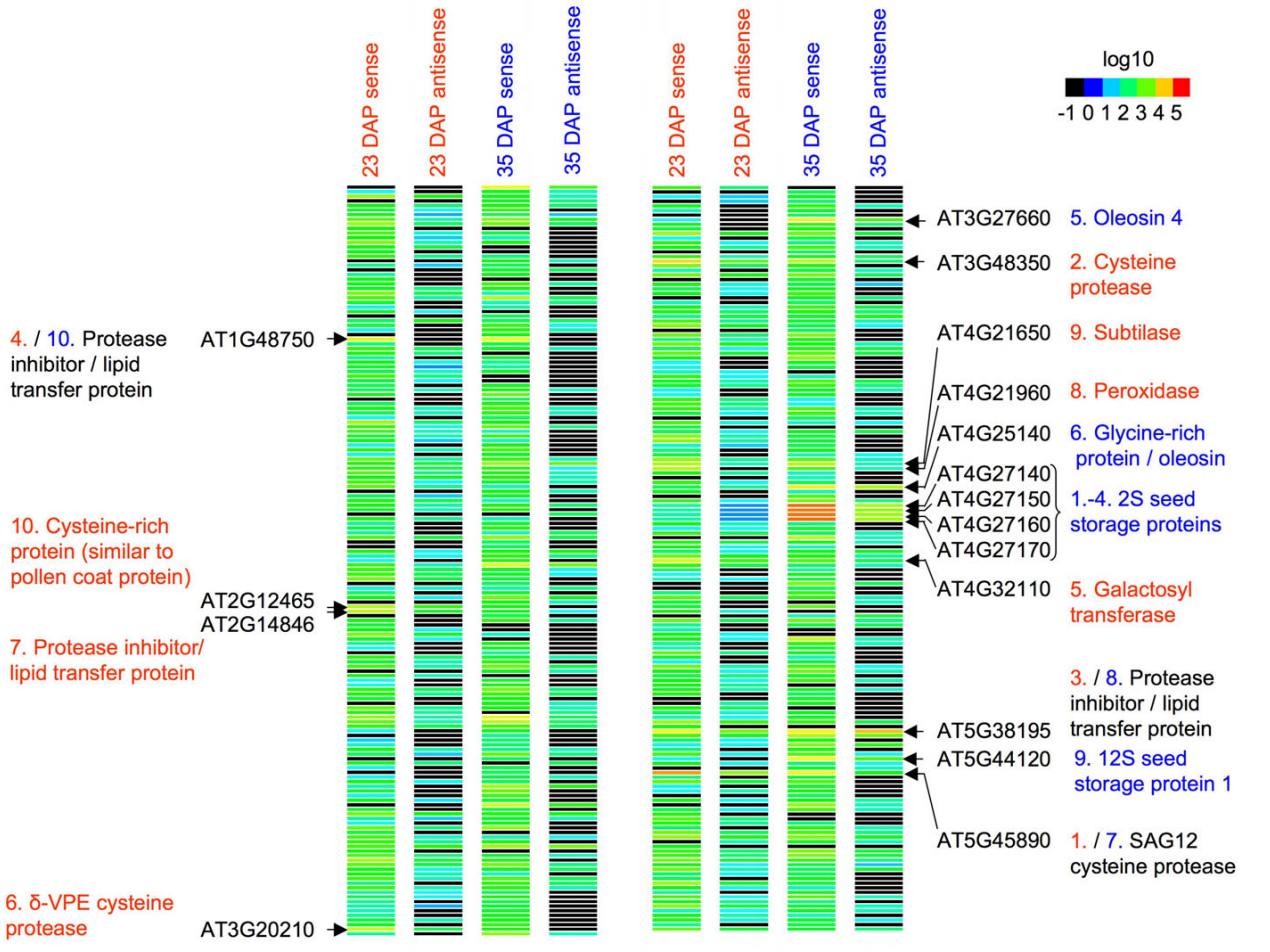
**Heat map of normalized LongSAGE counts for 375 differentially expressed *A. thaliana *genes matched by LongSAGE tags in sense and/or antisense orientation at 23 DAP and 35 DAP**. Expression is represented on a logarithmic scale. The descriptions of the 10 most abundantly expressed genes in the respective libraries are highlighted in red for 23 DAP, in blue for 35 DAP or in black for genes that were among the 10 most abundant at both time-points. If no expression was detected counts were set to 0.1 (black squares).

Based on the tag-to-gene matching strategy described above it was found that 24.3% of all matched tags matched *A. thaliana *genes via oriented *Brassica *ESTs in antisense orientation (corresponding to 1762 of 5,120, 34.4% of all matched genes, Table 2). In the 23 and 35 DAP libraries, only 68.3% and 74.6% of the respective genes were matched exclusively by sense tags (Table 2). On the other hand, 6.6% and 6.8% of all genes were matched exclusively by antisense tags at 23 DAP and 35 DAP, respectively, while 25.1% (23 DAP) and 18.6% (35 DAP) of the genes were matched by both sense and antisense tags. Genes expressed with high abundance in sense orientation, and particularly members of complex gene families, often also showed coexpression of antisense transcripts at a lower frequency (Figure 2, 275 differentially expressed genes matched in sense and antisense orientation). A detailed analysis of tags matching to the four 2S seed storage proteins of *A. thaliana *(AT4G27140, AT4G27150, AT4G27160, AT4G27170) was performed by aligning them to *Brassica *spp. napin cDNAs and genomic napin sequences. This revealed that some tags matched in antisense orientation to highly conserved regions of napin transcripts, derived from multiple *B. napus *loci, whereas other tags matched in sense orientation to highly diverse regions of napin transcripts derived from a limited number of *B. napus *loci.

### Detection of napin-related sense and antisense transcript expression by Real-time RT-PCR

At 35 DAP, a high number of tags matched in sense orientation to *Brassica *ESTs that were similar to *A. thaliana *proteins containing the AAI_SS domain (NCBI Conserved Domain Database cd00261). This protein domain is found in the alpha-amylase inhibitors (AAIs) and seed storage (SS) protein subfamily including plant lipid transfer proteins, seed storage proteins and trypsin-alpha amylase inhibitors. At 35 DAP the most abundantly expressed tags were matched via *Brassica *ESTs in sense orientation to the four *A. thaliana *2S seed storage proteins 1–4 (napins) AT4G27140, AT4G27250, AT4G27160 and AT4G27170 (37,182 to 41,806 tags per million). The four *A. thaliana *2S seed storage proteins share 75–91% amino acid (aa) sequence identity with each other. The four 2S seed storage protein genes and 7 other protease inhibitor/seed storage/lipid transfer protein (LTP) family protein genes were also among the genes with the highest tag counts matched in antisense orientation (see additional file 3: All_matched_tags.xls).

**Figure 3:**
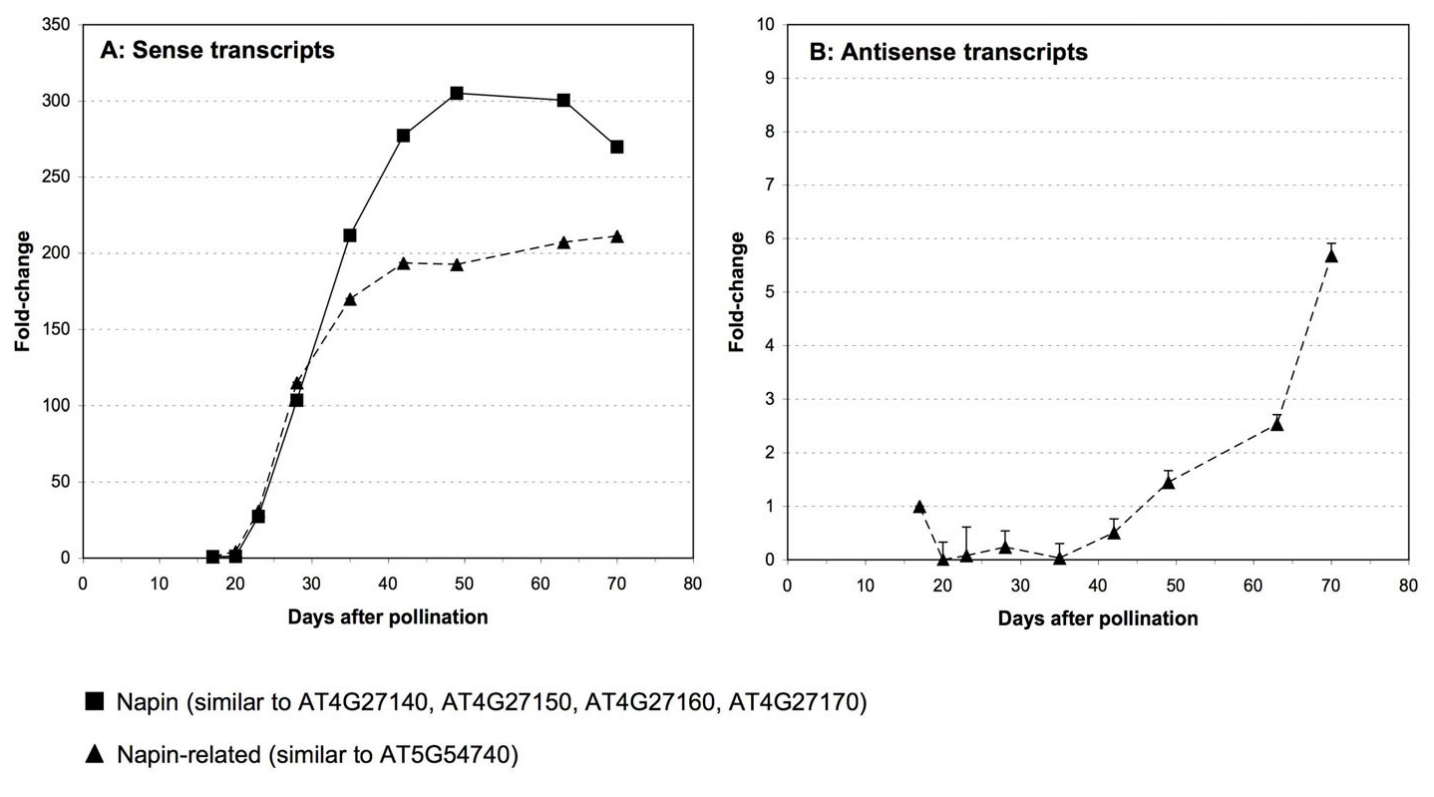
**Real-time RT-PCR expression profile during *B. napus *seed development using primers specific for the coding (A – sense transcripts) and non-coding strands (B – antisense transcripts) of a group of napin and napin-related transcripts**. Square symbols represent napin transcripts most similar to 2S seed storage proteins AT4G27140, AT4G27150, AT4G27160, AT4G27170, while triangular symbols represent napin-related transcripts most similar to protease inhibitor/seed storage/lipid transfer protein AT5G54740.

For comparison of SAGE results with quantitative Real-time RT-PCR detection, two sets of primers and their antisense reverse-complemented sequences were derived from SAGE primer amplified napin 3'-termini (see above) and from assembled contigs of 8,977 *B. napus *ESTs aligning with an e-value cut-off of 1e-6 to *A. thaliana *2S seed storage protein 1 (AT4G27140). One primer set (napin) was designed specifically for the major cluster of *B. napus *assembled EST contigs most similar to the *A. thaliana *2S seed storage proteins 1–4 (59% to 70% on the aa level). Another set of primers (napin-related) was designed specifically for the minor cluster of *B. napus *assembled EST clusters grouping in-between the four *A. thaliana *2S seed storage proteins and the related protease inhibitor/seed storage/lipid transfer (LTP) family protein AT5G54740 (about 47% to 57% identity on the aa level). AT5G54740 represents the next closely related protease inhibitor/seed storage/lipid transfer protein (LTP) family protein gene in *A. thaliana*, which like other more distantly related proteins of this large family (about 113 members in *A. thaliana*) exhibits an AAI_SS domain and shares about 53–57% aa identity with the *A. thaliana *2S seed storage proteins 1–4. Figure 3 shows the expression profile from days 17 to 70 after pollination from seeds for sense and antisense napin and napin-related transcript expression. The coexpression of sense and antisense transcripts was confirmed for a minor subgroup of napin-related transcripts by strand-specific Real-time RT-PCR, but not for the major napin transcript group as indicated by LongSAGE.

## Discussion

*B. napus *and *A. thaliana *are members of the Brassicaceae family and exhibit around 87% conservation of their protein-coding sequences [22]. Based on this close phylogenetic relationship, the tag-to-gene matching strategy applied in this study for LongSAGE expression profiling in the non-model plant *B. napus *uses the information available for the well-annotated proteome of the model species *A. thaliana*. Our results show that expression profiling can be achieved for *B. napus *seeds using publically available EST resources and the annotated proteome of *A. thaliana *for tag-to-gene assignment.

Major factors influencing the accuracy of the first step of the SAGE analysis are the sequencing error rate, the tag length and the transcriptome complexity. The accuracy of SAGE depends on the ability to unambiguously match the tags to the genes of origin. Theoretical calculations show that >99.8% of 21 bp LongSAGE tags are expected to occur only once in large genomes such as the human genome, which is about twice the size of the *B. napus *genome, with the remaining tags matching duplicated genes or repeated sequences [9]. In a polyploid plant genome the proportion of duplicated genes is generally considerably higher, and our strategy of matching tags via ESTs to a related model plant proteome will not *per se *allow differentiation between closely related duplicated genes. However, matching to multiple members of gene families still allows biological inferences from the LongSAGE data, since the predicted function in each case is identical. In our case only around 90% of LongSAGE tags were assigned to a unique *A. thaliana *gene. This underlines the highly duplicated nature of the *B. napus *and *A. thaliana *genomes [17] and reflects the well-documented ancient hexaploidization events in major angiosperm phyla [23]. Despite this we found no example where a *B. napus *tag matched multiple *A. thaliana *genes with apparently unrelated functions. This suggests that the matching strategy we applied is specific enough with 21 bp *B. napus *tags to draw biological conclusions on the expression levels of functionally related gene families in *B. napus *seeds. Raising the blastx e-value stringency to 1e-100 for matching of *Brassica *ESTs to the *A. thaliana *proteome was found to reduce the average number of genes per gene family to which the observed tags were assigned. However, this also resulted in a higher number of tags that could no longer be assigned to *A. thaliana *genes and thus could not be annotated (data not shown). For LongSAGE analysis from *B. napus *we found an e-value of >1e-6 useful; this is also the cut-off value used by NCBI in the UniGene database for EST-to-protein matching to create clusters of transcript sequences that are expected to come from the same transcription locus.

The development of oilseeds can be broadly divided into three phases, (1) the pattern formation/cell proliferation phase, (2) the maturation phase, with lipid and storage protein accumulation, and (3) the desiccation phase. At 23 DAP *B. napus *seed has reached the end of phase 1 and is beginning the transition to phase 2, while at 35 DAP the seed is in the middle of the maturation phase. The most abundantly expressed gene detected at 23 DAP, SAG12, is a cysteine protease expressed during leaf senescence in *A. thaliana *[24]. In *B. napus *SAG12 is encoded by two orthologous copies, BnSAG12-1 and BnSAG12-2, involved in leaf senescence and seed development, respectively. BnSAG12-2 and BnCysP1, a cysteine protease with around 91% identity to BnSAG12-2, are expressed in the early phase of *B. napus *seed development within the inner integument of the testa and are predicted to function in the disposal of proteins in the inner integument cells committed to programmed cell death [25]. Other genes encoding proteases and protease inhibitor genes are also strongly represented within the 30 most abundantly expressed genes at 23 DAP and 35 DAP, e.g. a δ-vascular processing enzyme (δ-VPE) at 23 DAP. VPEs are cysteine-type proteases which were originally discovered to be responsible for maturation of seed storage proteins. In *A. thaliana *δ-VPE was found to be involved in developmental cell death during embryogenesis [26]. In general, proteases are considered to be key regulators of plant development involved in post-translational modification or activation of proteins and enzymes of diverse biological processes. Proteases play a role both in general protein turnover and in highly specific regulation of plant development. Dong *et al*. [27] also described an elevated expression of genes coding for subtilisin-like protease and vacuolar processing enzymes (VPE), consistent with seed endosperm development, along with early expression of lipid transfer proteins that are required for embryo pattern formation [28,29]. The high transcription levels of genes encoding seed storage proteins are also consistent with the results of Dong *et al*. [27] based on cDNA cloning and Northern blot expression profiling. On the other hand, our LongSAGE analysis revealed that transcripts encoded by the peroxidase gene AT4G21960 (Protein ID CAA66957) had a high abundance at 23 DAP and a subsequent drop in expression at 35 DAP, whereas Northern blot analysis appeared to reveal the opposite expression pattern. Such differences might be due to different specificities and resolutions of the two techniques, e.g. cross-hybridization of Northern blot probes with other closely related peroxidase transcripts.

Antisense transcripts and sense-antisense transcript pairs (natural antisense transcripts, NATs) were found to be prevalent at both developmental stages investigated. Antisense transcripts were observed earlier in *A. thaliana *and rice cDNA/EST databases and confirmed using tag-based expression profiling approaches like SAGE or Massively Parallel Signature Sequencing [30], with matching to the sequenced genomes. In some of these studies it was noted that the sense and antisense transcripts of an overlapping NAT pair (*cis*-NATs) tend to be expressed in different tissues or different conditions. Furthermore, in cases where the sense and antisense transcripts of a NAT pair were expressed in the same library, one type of transcript was usually more abundant than the other. One speculation is that *cis*-antisense transcripts could be involved in down-regulating the expression levels of their target mRNA to achieve a low protein concentration by interfering with the transcription of their sense transcript. Due to the matching strategy we applied, the sense-antisense pair matches we found to the *A. thaliana *proteome might either indicate natural sense-antisense pair expression from one *B. napus *gene, or a concurrent expression of sense and antisense transcripts from paralogous *B. napus *genes. Either way, the results clearly indicate that a significant number of antisense transcripts are expressed during *B. napus *seed development. The coexpression of sense and antisense transcripts was confirmed for a subgroup of napin-related transcripts coding for proteins from the large group of protease inhibitor/seed storage/lipid transfer protein (LTP) family by strand-specific Real-time RT-PCR. Although Real-time RT-PCR confirms the detection of antisense transcripts that are related to protease inhibitor/seed storage/lipid transfer protein (LTP) family proteins by LongSAGE, it also indicates that the use of the proteome of *A. thaliana *for annotation of tag-matched *B. napus *transcripts in case of diverse gene families limits the resolution and the detailed analysis of the genome and transcriptome structure and availability of more genetic resources for *B. napus *will help to increase the resolution in the future. The detection of transcripts for a diverse number of genes in antisense orientation by LongSAGE at both time-points suggests strong involvement of antisense transcripts in regulatory processes during *B. napu*s seed development.

The comparison of LongSAGE and Real-time RT-PCR data suggests that the sequencing depth applied in this experiment limits the LongSAGE detection to transcripts with medium to high copy numbers in *B. napus *seeds. On the other hand, recent developments in next-generation sequencing (NGS) technologies allow ultra-deep transcriptome profiling using transcript tag-based techniques (e.g. DeepSAGE; [31]) that can realistically achieve transcriptome saturation and accurate quantification of low-copy transcripts. The data analysis techniques developed in the present study represent a valuable platform for future NGS-based transcriptome tagging studies in *Brassica *species. As suggested by Shendure [32], such approaches may ultimately replace microarrays as the method of choice for quantitative global transcriptome profiling as a basis for genetical genomics or systems genetics approaches. In the present study we have demonstrated that tag-based transcriptome profiling can also be effectively applied in a non-model, complex polyploid plant species.

## Conclusion

This study underlines the potential of transcript tagging approaches for gene expression profiling in *Brassica *crop species via EST matching to annotated *A. thaliana *genes. The data processing pipeline adapted by matching *B. napus *tags via *Brassica *ESTs to annotated *A. thaliana *gene loci enabled differential expression profiling of during seed development in the complex *B. napus *genome, and furthermore detected an unexpectedly high proportion of EST tags matching in antisense orientation. This suggests a strong involvement of antisense transcript expression in regulatory processes during *B. napu*s seed development. Limits of tag detection for low-abundance transcripts can today be overcome by ultra-high throughput sequencing approaches, so that tag-based gene expression profiling may soon become the method of choice for global expression profiling in non-model species.

## Methods

### Plant materials and RNA isolation

Plants of the homozygous male-sterile winter oilseed rape maternal line 'MSL-Express' were cultivated under controlled growth chamber conditions (16 hour, 20°C day and 8 hour, 16°C night, 60% relative humidity) and manually fertilised with pollen from plants of the isogenic male-fertile line 'Express 617'. Pods were harvested weekly from the main racemes of pollinated plants at time-points 14 days up to 70 days after pollination (DAP). The pods were shock-frozen in liquid nitrogen and stored at -80°C, and seeds were collected by splitting the frozen pods. Total RNA was extracted from seeds using RNA Reagent (Invitrogen) and purified from DNA residues by incubation with RQ1 RNAse-free DNAse (Promega), according to the manufacturer's recommendations.

### LongSAGE library production

Total RNA extracted from two seed developmental time-points, 23 DAP and 35 DAP, was processed using the I-SAGELong Kit (Invitrogen) with several modifications to allow more efficient library production and cloning. Briefly, 50 μg of polyA+ RNA was captured on the surface of magnetic oligo(dT) beads and double stranded cDNA was synthesized using SuperScript II reverse transcriptase. Double stranded cDNA was digested for 2.5 hours with *Nla*III, and adapters containing *Mme*I recognition sites were ligated overnight in ligation buffer with 15% polyethylene glycol (PEG) to the 5' end of the cDNAs. Adaptor-linked tags were released from the beads by restriction with *Mme*I overnight, then precipitated and washed five times with 70% ethanol before being ligated overnight in pairs to form adaptor-linked ditags. These were amplified by PCR and 200 PCR reactions were pooled. Subsequently, 130 bp fragments were purified from 20 lanes on 16% polyacrylamide gels. Purified adapter-linked ditags were redigested overnight with *Nla*III to remove adapters and 34 bp ditags were purified from a 16% polyacrylamide gel. The ditags were ligated overnight according to Kenzelmann and Mühlemann [33] to form concatemers, then partially digested with 2 U of *Nla*III for 1 min at 37°C according to Gowda *et al*. [34] to create linearized clonable concatemers of suitable sizes. The partially-digested ligation mixture was separated on a 6% polyacrylamide gel. DNA fragments between 0.8 and 2.5 kb were isolated and 1.6 μg concatemers were ligated to 25 ng of linearized pZErO-1 vector digested with *Sph*I. For each of the 23 and 35 DAP libraries more than 3,000 plasmid clones were sequenced in one direction by SeqWright (Houston, TX, USA).

### SAGE data processing

Ditags were extracted from sequence traces using the perl script sage-prhed.pl [35]. Due to the 1% error rate associated with single pass Sanger sequencing [14], Phred sequence trace quality scores were used to remove all tags of low sequence quality (containing bases with a Phred score <20). 21 bp monotags were extracted from both ends of all correctly-sized ditags. Contaminating linker and vector sequences and low-complexity polyA tags were identified and removed by screening a local tag database using formatdb and blast (valid tags in Table 1; <0.06% removed). A total of 819,455 *Brassica *ESTs were downloaded on Sept, 15^th ^2007, from NCBI dbEST  using the keyword search (Brassica napus [Organism] OR Brassica oleracea [Organism] OR Brassica rapa [Organism] AND EST [Keyword]). About 30% of all *Brassica *ESTs downloaded from the NCBI dbEST database were produced from seed developmental stages. The orientation of all *Brassica *ESTs was determined by blastx analysis against the TAIR7 *A. thaliana *proteome accepting only matches with an e-value of less then 1e-6. Any *Brassica *ESTs that did not match the *A. thaliana *proteome using these criteria were removed from the dataset. ESTs that did match the NCBI VecScreen vector database using blastn with pre-set parameters were removed from the analysis. The remaining 834,732 ESTs from NCBI and PBI were annotated based on the alignment with the *A. thaliana *proteome and orientated in sense using ReadSeq [36]. Identitag perl scripts were modified so that virtual tags from all positions were extracted from the *Brassica *ESTs, and the modified Identitag perl scripts were used to create a MySQL database of virtual tags [37]. To differentiate between tags that matched *B. napus *seed transcripts in sense and in antisense orientation, the oriented sense EST dataset was used in combination with an oriented reverse-complemented EST dataset (antisense EST dataset). For identification of differentially expressed tags the online-tool DiscoverySpace (; [38]) was used and significance calculated according to Audic and Claverie [39]. The online-tools WEGO (; [40]) and GOEAST (; [20]) were used for gene ontology (GO) enrichment analysis. Hierarchical cluster analysis with average linkage (UPGMA) and Euclidean distance was performed for GC-RMA processed, normalized data using the software program HCE 3.0 [41]. Spearman's rank correlations were used to compare Affymetrix chip and LongSAGE [19]. Differentially expressed genes were identified using the software program SAM [42].

### Cloning of 3'ends of transcripts using SAGE tag primers

For validation, the last 17 base pairs of observed LongSAGE tags (excluding the CATG restriction site) were used as primers for RT-PCR amplification together with anchored oligo-dT primers, as described by Xiao *et al*. [43], and 2.5 μg total RNA from time-point 35 DAP. cDNA synthesis was performed using ThermoX Reverse Transcriptase (Invitrogen) according to the manufacturer's instructions. The PCR used 0.5 U *Taq *Polymerase (Eppendorf) and 250 nM of each primer, with 2 min at 95°C followed by 30 cycles of 1 min 95°C, 1 min 50°C, 2 min 72°C and final extension at 72°C for 10 min.

### Strand-specific Real-time RT-PCR

Total RNA was extracted as described above and additionally purified using RNAeasy columns (Qiagen). DNase digestion was performed using RQ1 RNAse-free DNAse (Promega) according to manufacter's recommendations. cDNA was synthesized after denaturation for 10 min at 70°C using 1 μg of total RNA, 100 nM of gene and strand-specific reverse primer and Omniscript Reverse Transcriptase (Qiagen) according to the manufacturer's recommendations in a total volume of 10 μl. After the cDNA synthesis reaction was finished the enzyme was inactivated for 15 min at 95°C. Real-time PCR was performed using a 7500 Fast Real-time PCR system (Applied Biosystems) with a total volume of 10 μl using 1 μl of cDNA (1:10 diluted), 5 μl of Power SYBR Green PCR master mix (Applied Biosystems) and 100 nM each of the forward and reverse primers. Amplification was for 2 min at 50°C, 10 min at 95°C followed by 40 cycles of 15 sec at 95°C and 1 min at 60°C. Controls included PCR amplification reactions using cDNA preparations without adding the reverse transcriptase enzyme to check for genomic DNA contamination, and PCR amplification reactions without adding reverse primers during cDNA synthesis to check for a loss in strand-specificity by RNA self priming leading to primer-independent cDNA synthesis [44]. Primers were designed by using *Brassica *ESTs that were matched using blastx at an e-value cut-off of 1e-6 with selected *A. thaliana *proteins. All matched *Brassica *ESTs were assembled using the software CAP3 [45] and edited manually. Alignments of coding DNA were constructed from aligned amino acid sequences using the software RevTrans [46]. Contigs from these assemblies were used for primer design using the software PrimerExpress (Applied Biosystems). Primer pairs used for sense transcript detection were Napin_for (5'ACGAGCTCCACCAGGAAGAG-3') and Napin_rev (5'-ACGGCTTTGGATGCTCCTT-3'), which amplify position 320 to 383 relative to the coding region of gene napB [GenBank:X14492], and Napin-related_for (5'-AACAAGCAGCCAAGTCAGTTAGG-3') and Napin-related_rev (5'-GATGCGGGTGGACTGGAAT-3') which amplify position 323 to 384. For antisense transcript detection the reverse complemented sequences of the primers were used. Expression fold-changes were calculated relative to the endogenous reference gene cyclophilin (GenBank accession M55018) according to the 2^-ΔΔCt ^method using three replicates [47].

## Authors' contributions

RS conceived the project and WF provided the plant material. CO and BH generated the SAGE libraries. CO adapted and applied bioinformatic tools for analysis of LongSAGE tags. CO designed the primers for Real-time RT-PCR and BH performed the experiments. CO and RS developed the experimental strategy, and were responsible for the preparation of the manuscript. RS and WF participated in the coordination of the study. All authors read and approved the final manuscript.

## Supplementary Material

Additional file 1 **490 differentially expressed genes between 23 and 35 DAP**. Description and normalized counts for 490 loci matched by LongSAGE that were differentially expressed in *B. napus *seeds at 23 or 35 days after pollination (DAP). Click here for file

Additional file 2 **Enriched GO terms for 490 differentially expressed genes between 23 and 35 DAP**. Statistically enriched Gene Ontology (GO) terms exclusively found for one of the time-points 23 DAP and 35 DAP of *B. napus *seed development (p < 0.05) for 490 differentially expressed genes calculated using GOEAST (Zheng and Wang, 2008). Click here for file

Additional file 3 **All LongSAGE tags extracted from *B. napus *seed at 23 and 35 days after pollination (DAP) and matched via Brassica ESTs to *A. thaliana *genes**. Description and normalized counts for all *A. thaliana *loci matched by LongSAGE that were expressed in *B. napus *seeds at 23 or 35 days after pollination (DAP) and matched in sense and/or antisense orientation. Genes are sorted by locus name. Click here for file
